# Which femoral neck for a dual mobility cup? A biomechanical evaluation

**DOI:** 10.1007/s00264-022-05415-z

**Published:** 2022-05-16

**Authors:** Julien Wegrzyn, Jason Longaray, Rafael Baez, Lizeth Herrera

**Affiliations:** 1 Department of Orthopedic Surgery, Lausanne University Hospital and University of Lausanne, Avenue Pierre Decker, 4, CH – 1011 Lausanne, Switzerland; 2 Stryker, Mahwah, NJ USA

**Keywords:** Dual mobility cup, Third articulation, Femoral neck, Impingement, Polyethylene wear

## Abstract

**Purpose:**

This study aimed to evaluate polyethylene (PE) damage and wear lesions to the chamfer of mobile components under mobile and fixed femoral neck impingement at the third articulation, and to determine which femoral neck characteristics should be considered with a dual mobility cup to limit those lesions.

**Methods:**

Two femoral neck geometries (cylindrical and quadrangular) with two surface finishing roughness (rough and polished), and two head-to-neck ratios (28- and 22.2-mm diameter femoral heads) were evaluated in a hip simulator testing. For each characteristic, six femoral necks were tested with six dual mobility cups under fixed and mobile femoral neck impingement conditions. Chamfer PE damage and volumetric wear were evaluated and compared for each femoral neck characteristic and impingement condition.

**Results:**

Under mobile impingement condition, femoral neck characteristics did not significantly affect PE damage and wear lesions to the chamfer (*p* = 0.283 to 0.810). However, under fixed impingement condition, significantly higher PE damage and wear lesions to the chamfer were produced by the quadrangular geometry compared to the cylindrical geometry (*p* = 0.004 to 0.025). In addition, with the quadrangular geometry, rough surface finishing was demonstrated to increase volumetric wear of the chamfer (*p* = 0.009). No significant influence of head-to-neck ratio was observed on PE damage and wear lesions to the chamfer (*p* = 0.244 to 0.714).

**Discussion:**

This biomechanical study emphasized that femoral neck characteristics are critical with dual mobility cup and tend to favor a cylindrical geometry particularly whether fixed impingement at the third articulation occurs.

## Introduction

**Fig. 1:**
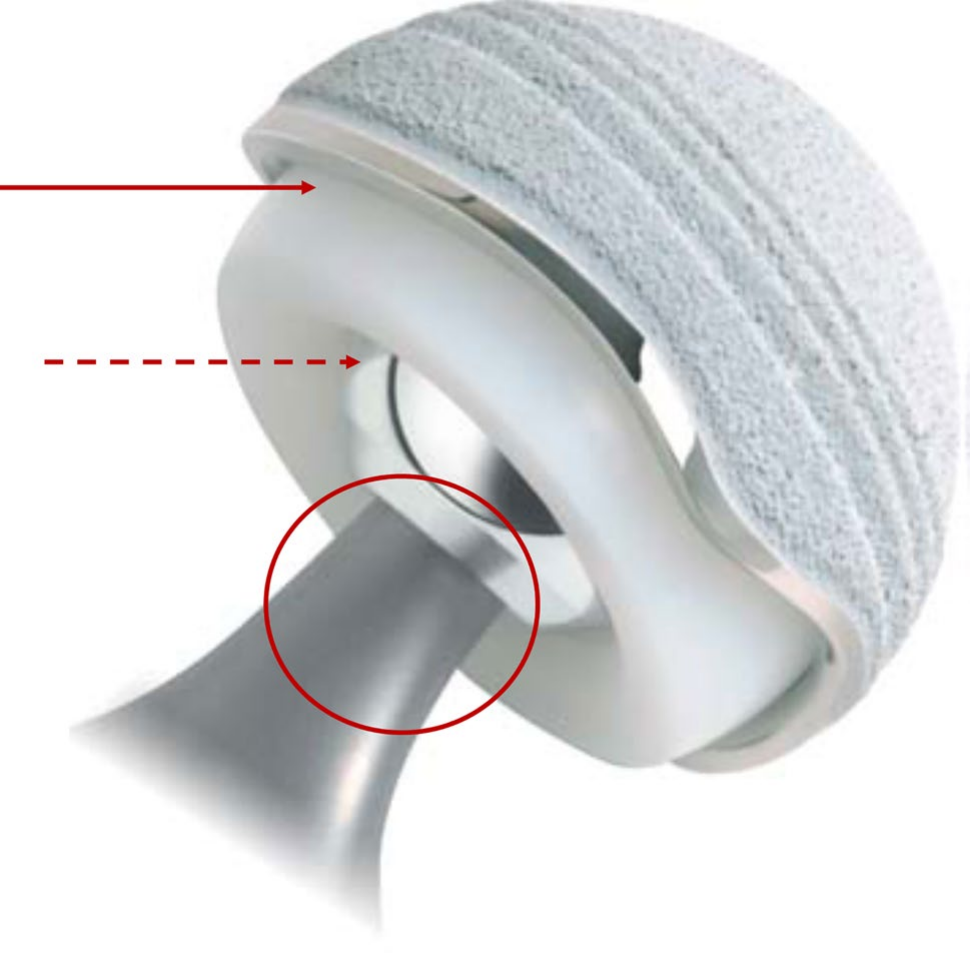
Dual mobility cup construct with ADM® cup and Accolade2® stem (Stryker, Mahwah, NJ). The plain arrow illustrates the large articulation, the dashed arrow the small articulation, and the circle the location of the third articulation

The principle of dual mobility cup (DMC) relies on three prosthetic articulations with the “small articulation” between the polyethylene (PE) mobile component and the femoral head that results in a low-friction inner bearing, and the “large articulation” between the mobile component outer surface and the metal-shell that results in a large PE-head outer bearing (Fig. 1) [1–4]. A “third articulation” was described by Noyer between the mobile component chamfer (i.e. the outer surface of retentive area) and the femoral neck that allows the mobile component to displace at the large articulation upon femoral neck contact with minimal resistance in a well-functioning DMC (Fig. 1) [3, 4]. Lecuire et al. first emphasized the critical importance of the third articulation in the occurrence of intraprosthetic dislocation (IPD) related to long-term wear of the mobile component chamfer and retentive area [5–7]. Retrieval and biomechanical studies further demonstrated that motion and wear predominated at the small and third articulations with potential PE damage and deformation onto the chamfer due to femoral neck impingement, particularly in case of restricted motion at the large articulation [5–15]. In a well-functioning DMC, Nebergall et al. and Loving et al. reported that, as the mobile component freely moves along three axes, a mobile impingement of the femoral neck occurred onto the mobile component chamfer at the third articulation [14, 15]. In this condition, PE damage and wear lesions were observed to be circumferentially spread throughout the mobile component chamfer and retentive area [15]. Conversely, in case of restricted motion at the large articulation and for a given amount of load transferred to the third articulation by the femoral neck, PE damage and wear lesions would be likely affected and concentrated in areas where the fixed impingement occurs [6, 14, 15]. Therefore, although frequently under-considered in literature evaluating DMC, the femoral neck characteristics could affect PE wear and damage lesions onto the mobile component chamfer at the third articulation. However, despite general recommendations regarding those characteristics issued from clinical series, no biomechanical evaluation of the impingement between the femoral neck and the mobile component chamfer at the third articulation has been performed to determine the optimal characteristics of a stem to be used with a DMC [4–10].

Therefore, this biomechanical study aimed (1) to evaluate the PE damage and wear lesions to the mobile component chamfer under fixed and mobile femoral neck impingement conditions, and (2) to determine which characteristics of the femoral neck (i.e., geometry, surface finishing roughness, and head-to-neck ratio) should be considered in total hip arthroplasty (THA) with DMC in order to limit those PE lesions potentially leading to critical wear at the third articulation.

## Material and methods

### Implants and femoral neck characteristics

**Fig. 2:**
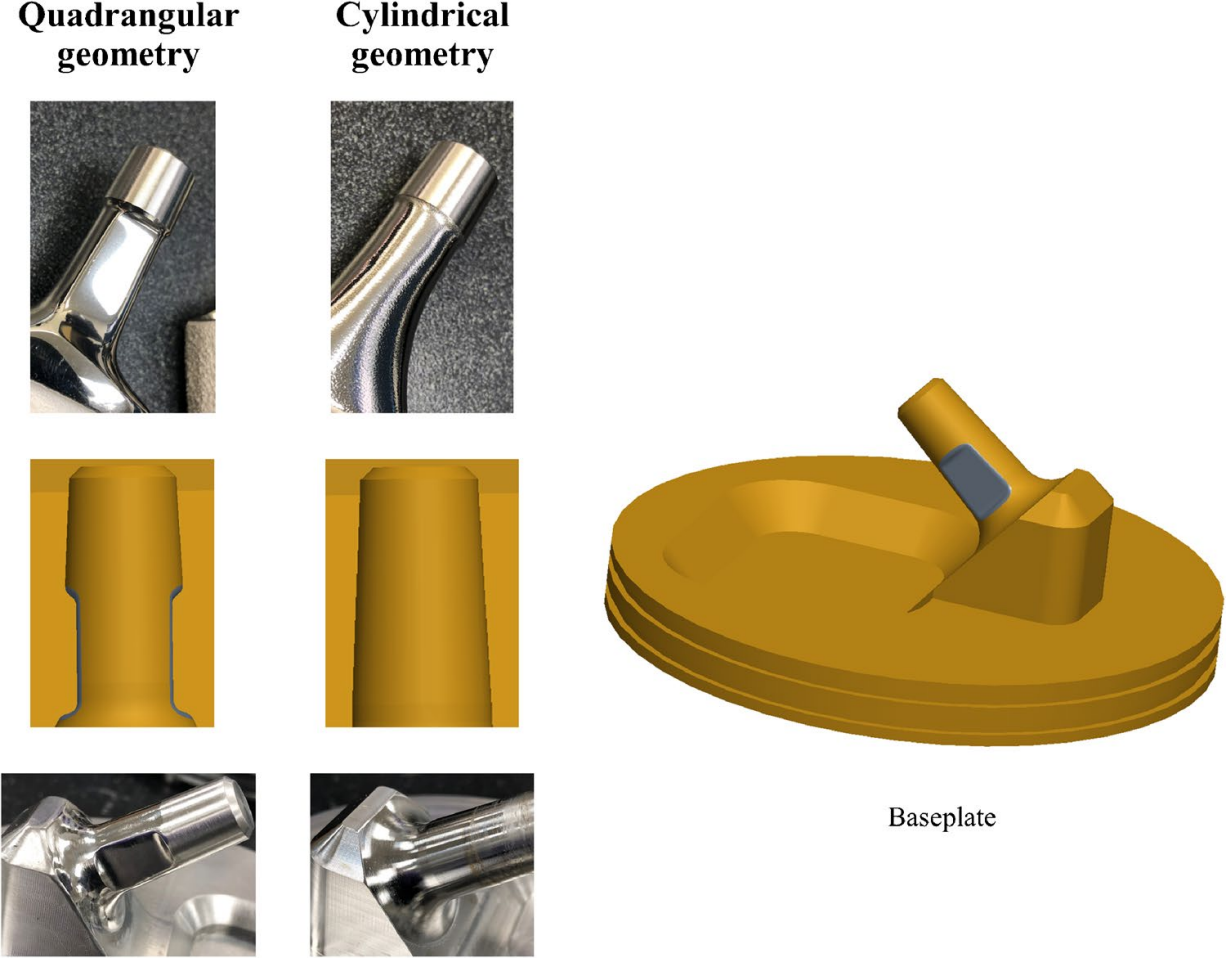
Photographs and schematic representations of the two geometries of femoral neck (i.e., cylindrical and quadrangular)

**Fig. 3:**
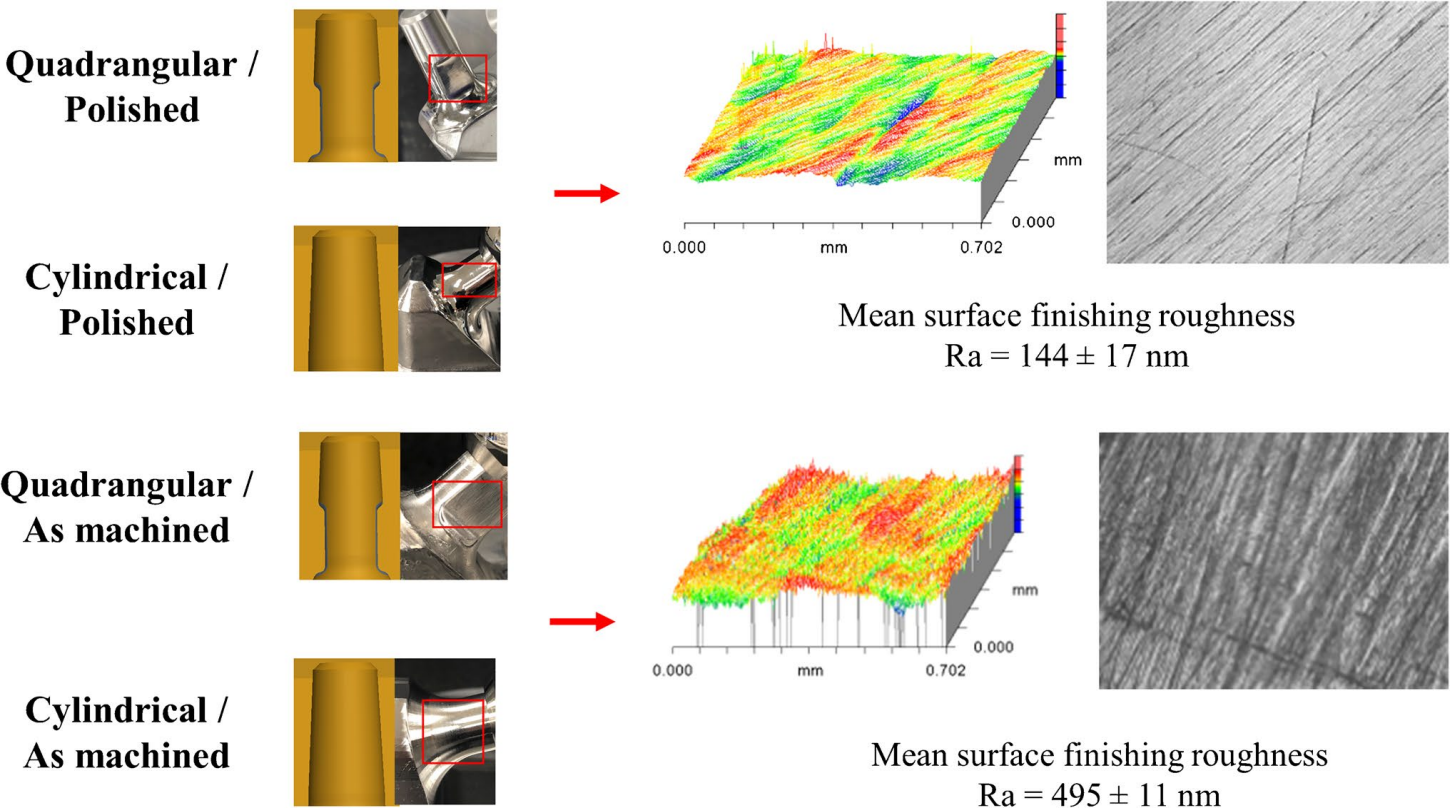
Photographs, schematic representations, and surface analysis illustrating the two surface finishing roughness of femoral neck

Two different geometries of femoral neck with two different surface finishing roughness and two different head-to-neck ratios were evaluated. The femoral necks were custom-made from 316-L stain steel blocks (Fig. 2). Using reverse engineering machining from commercially available implants, the two femoral neck geometries were (1) quadrangular with rounded corners and 12/14 mm Morse taper, and (2) cylindrical with V40 (11/13 mm) Morse taper (Fig. 2). For each geometry, the two surface finishing roughness were (1) rough “as-machined” and (2) smooth “polished” (Fig. 3). The mean surface finishing roughness was assessed for each femoral neck with three measurements around the neck using a white light interferometer (NewView 6300®, Zygo, Middlefield, CT) with a resolution of 0.1 nm. The mean average roughness was 144 ± 17 nm for the “polished” surface finishing and 495 ± 11 nm for the rough surface finishing. The head-to-neck ratio was defined according to the cobalt-chromium (Co-Cr) femoral head diameter (i.e., 28- or 22.2-mm diameter, head offset = 0).

For each characteristic of geometry and surface finishing roughness, six femoral necks were tested in front of six ADM® DMC (Stryker, Mahwah, NJ) under mechanical conditions of either a mobile or fixed femoral neck impingement at the third articulation simulating in vitro mechanical conditions of either a well-functioning DMC or DMC with restricted motion at the large articulation such as observed in case of arthrofibrosis (Table 1) [5–7, 15]. For the head-to-neck ratio comparisons, only a rough surface finishing was tested for both geometries under fixed impingement as these conditions were considered as the worst-case scenario (Table 1). The DMC metal shell was 54-mm outer and 48-mm inner diameters with a highly polished CoCr bearing surface. The mobile components were made of compression molded GUR 1020 ultra-high molecular weight PE (UHMWPE) sequentially irradiated and annealed three times at a dosage of 30 kGy each time for a total dosage of 90 kGy resulting in a second-generation highly cross-linked PE (X3®, Stryker, Mahwah, NJ). The mobile components were designed for either a 28- or 22.2-mm diameter femoral head (Table 1).

**Table 1:** Description of the hip simulator impingement testing with the different combinations of implants, femoral neck characteristics (i.e., geometry, surface finishing roughness, and head-to-neck ratio), and impingement conditions

Femoral neck characteristics	Impingement condition	Implant description
Cylindrical rough and polished 28-mm diameter femoral head	Mobile and fixed	ADM® with X3 mobile component 28/54 mm
		28 mm + 0 Co-Cr femoral head
		*N* = 6 in the 4 groups
Quadrangular rough and polished 28-mm diameter femoral head	Mobile and fixed	ADM® with X3 mobile component 28/54 mm
		28 mm + 0 Co-Cr femoral head
		*N* = 6 in the 4 groups
Cylindrical rough 22.2-mm diameter femoral head	Fixed	ADM® with X3 mobile component 22.2/54 mm (custom-made)
		22.2 mm + 0 Co-Cr femoral head
		*N* = 6
Quadrangular rough 22.2-mm diameter femoral head	Fixed	ADM® with X3 mobile component 22.2/54 mm (custom-made)
		22.2 mm + 0 Co-Cr femoral head
		*N* = 6

### Impingement conditions and testing

**Fig. 4:**
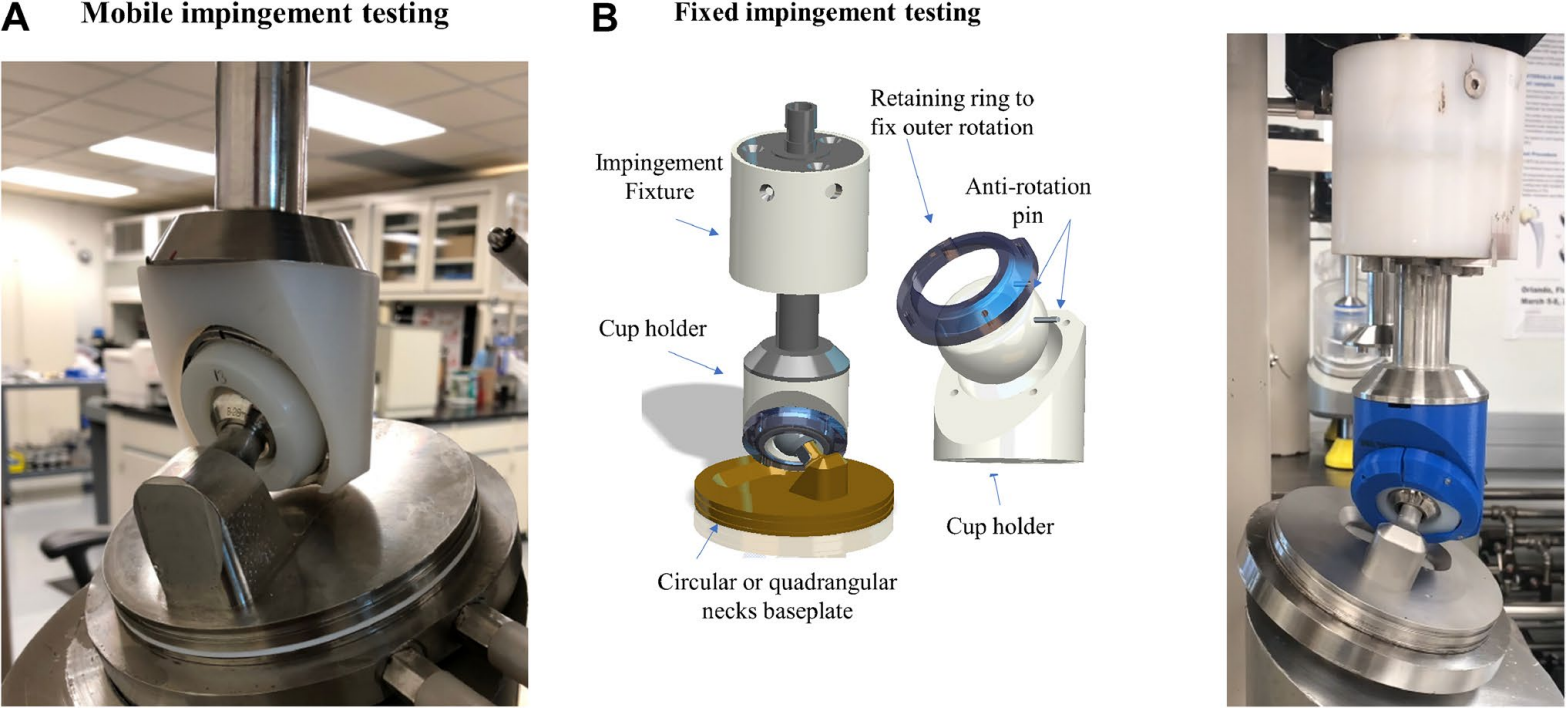
(**A**) Hip simulator mounting with the ADM® dual mobility cup (Stryker, Mahwah, NJ) and custom-made machined cylindrical femoral neck construct for mobile impingement testing. (**B**) Hip simulator mounting and retaining system of the polyethylene mobile component for fixed impingement testing

The impingement testing were performed under two conditions: (1) a mobile impingement condition with an unconstrained mobile component at the large articulation simulating a well-functioning DMC and (2) a fixed impingement condition with the mobile component being immobilized by the fixture simulating a DMC with restricted motion at the large articulation (Fig. 4) [1–4, 13, 15]. All the testing were conducted on a multi-joint hip wear simulator (MTS®, Eden Prairie, MN) for each femoral neck characteristic and impingement condition for a total of 1.0 million cycles (mc) that corresponds to one year of normal activity [16–18].

**Fig. 5:**
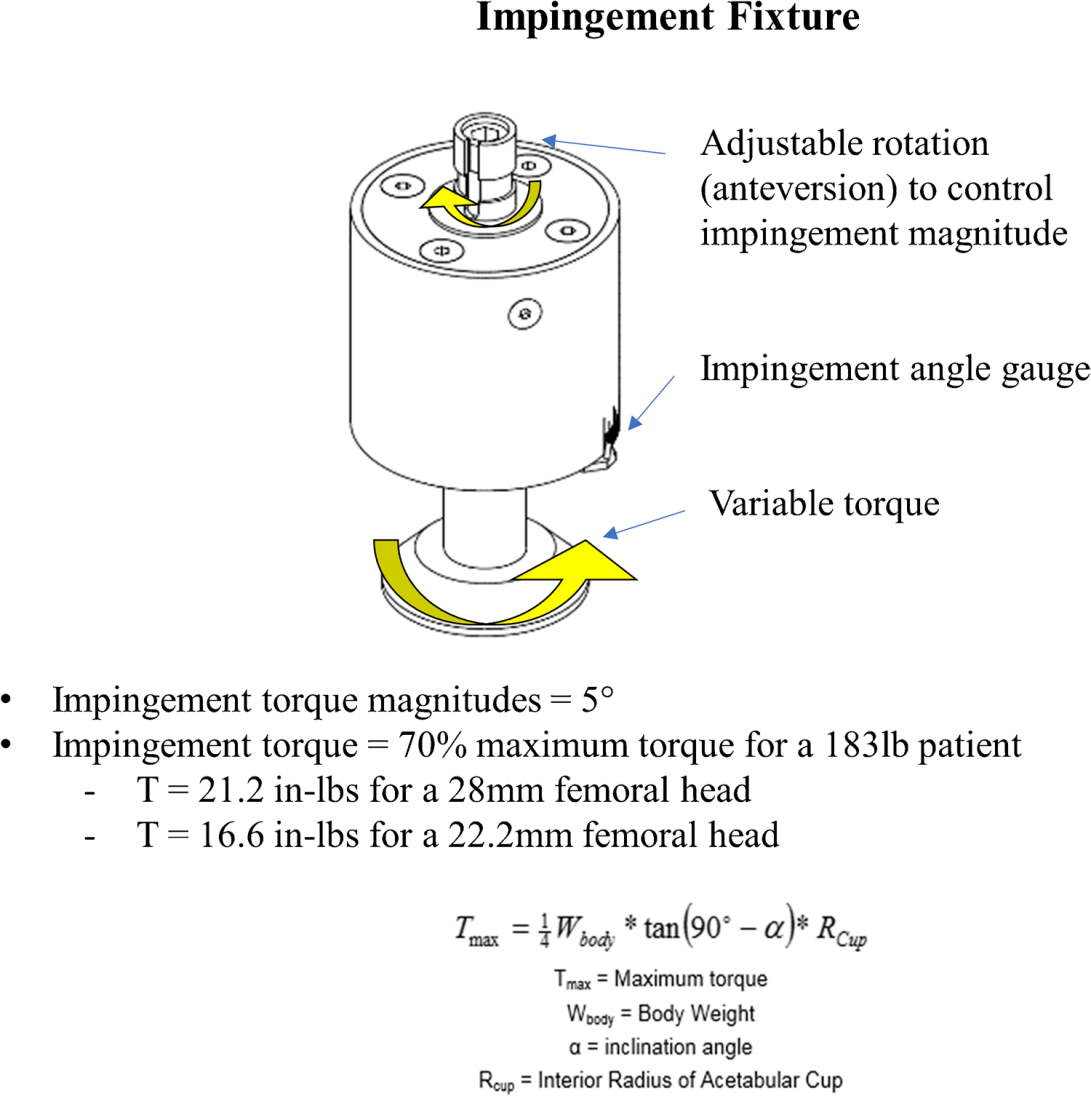
Schematic representation of the impingement testing controllable torque system

In the hip simulator set-up, stationary acetabular components were mounted superiorly to femoral necks along the same vertical axis. Each block supporting the femoral neck was fixed to an inclined baseplate at 23° rotating at 1 Hz to provide a composite flexion/extension, abduction/adduction, and internal/external rotational motion [16]. This angle was chosen to represent human level walking gait and has been based on previously developed ISO 14242–3:2009 protocols [19]. Compressive loading was applied axially with a maximum of 2450 N following the physiological walking gait curve profile determined by Paul et al. [20]. To create the mobile impingement condition, the femoral neck was positioned at 17° of anterversion and the metal-shell was fixed at 45° of inclination with neutral anteversion [15]. At these angles, the impingement with the mobile component occurred at either the superior or inferior surface of the femoral neck during the walking gait cycle. After the first gait cycle, the unconstrained nature of the mobile component allowed the articulation to run impingement-free for the majority of testing cycles with impingement occurring randomly and re-aligning itself repeatedly throughout the testing duration (Fig. 4A). To create fixed impingement condition, the mobile component was seated directly into the fixture at 45° of inclination with neutral anteversion, and then clamped down with a retaining ring and anti-rotational pin to only allow motion at the small articulation (Fig. 4B). The femoral neck impingement was applied onto the mobile component chamfer using a controllable torque system (Fig. 5). The system was controlled using a spring mechanism to induce variable levels of torque based on the desired impingement condition (Fig. 5). The mobile component and femoral neck positioning were controlled to produce a torque that created a joint force of approximately 25% of the body weight when impingement occurs (Fig. 5).

### Measurements and analysis

**Fig. 6:**
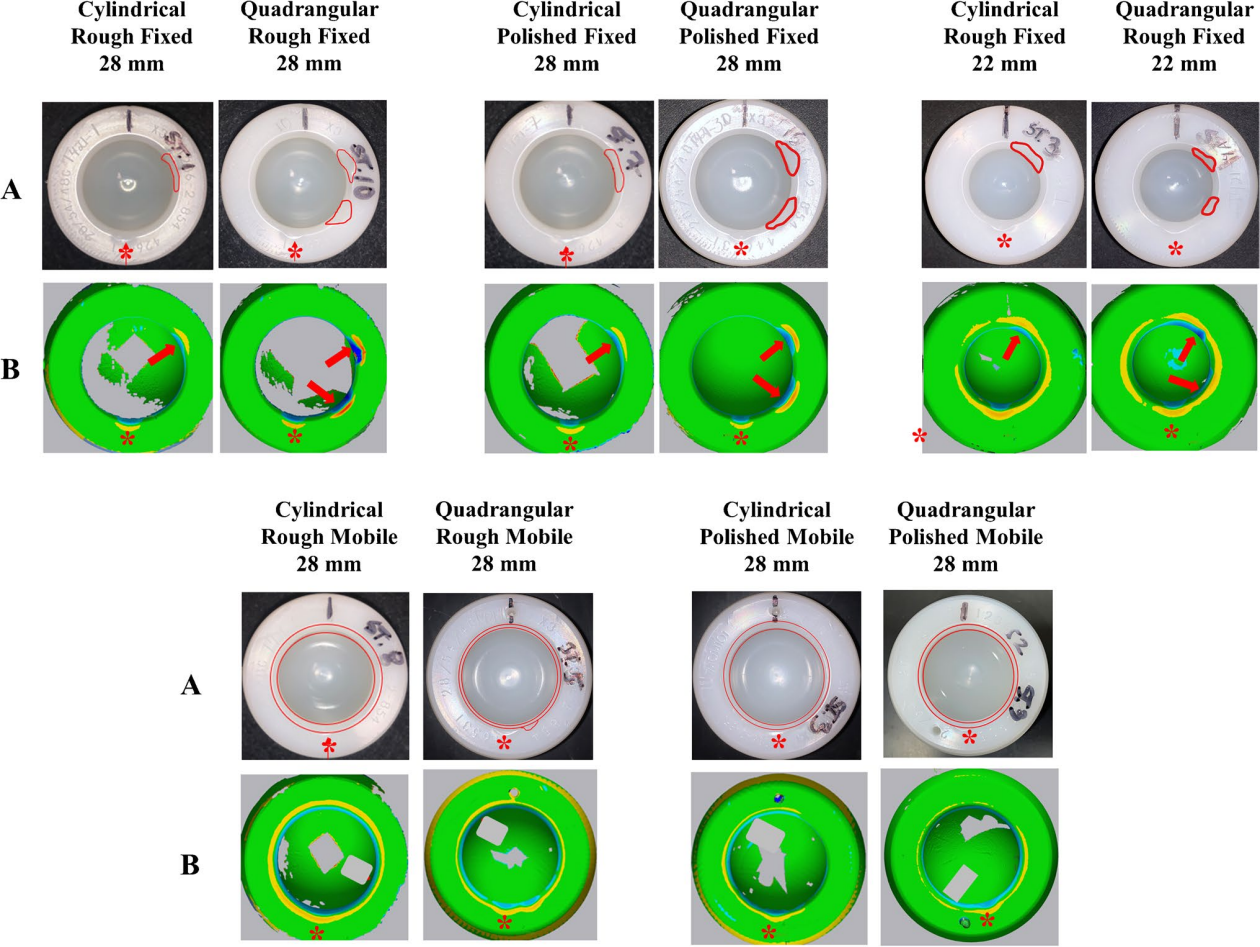
(**A**) Macroscopic visual analysis and (**B**) surface analysis using a blue light 3D surface scanning system of the mobile component chamfer after impingement testing (red arrows: location of the femoral neck impingement contacts and red star: location of the lever-out lesion after femoral head snap-out removal)

Prior to testing, all the mobile components were pre-soaked in deionized water for 21 days at a controlled-temperature of 37 °C. During testing, a chamber surrounded the construct and allowed for lubricant submersion of the mobile component into lubricant solution at a room temperature. The lubricant used was alpha calf serum (Hyclone Alpha Calf Fraction, Logan, UT), diluted to 50% using a pH-balanced 20-mmol solution of deionized water and ethylenediaminetetraacetic acid (EDTA) to obtain a physiological relevant protein level (approx. 20 g/dL). EDTA was added to retard serum decomposition and the solution was filtered through a 20-μm filter before use. All the fixtures were composed of non-corrosive materials. In addition, all the fixtures and components were ultrasonically cleaned prior to testing. Then, the testing was stopped after every 250,000 cycles for lubricant changing, component cleaning, and gravimetric wear measurements using an analytical microbalance (Sartorius®, Sartorius Corporation, Göttingen, Germany). Soaked mobile component controls were used to correct for net weight gain due to fluid absorption. Soaked-corrected gravimetric measurements were converted to volumetric wear by dividing by material density of PE. Linear regression of the volumetric wear versus cycle counts was used to determine the volumetric wear of mobile components in mm^3^ at 1.0 mc. To accommodate for gravimetric wear analysis, the femoral heads were snap-out removed from mobile components using a lever-out technique with particular attention to apply the lever-out force on a chamfer location that did not present any impingement lesion (Fig. 6). The lever-out lesion was carefully identified for each case. Each mobile component was macroscopically evaluated before and after testing to assess location and pattern of impingement lesion onto the chamfer due to femoral neck contact (i.e., single contact, two-point contact, or circumferential contact) (Fig. 6). In addition, surface analysis was performed using a blue light 3D surface scan (Geomagic Capture® Mini 3D, 3D Systems, Rock Hill, SC) allowing to measure in mm the impingement deformation depth onto the chamfer (i.e., maximal linear penetration of the femoral neck) due to femoral neck contact at 1.0 mc using Geomagic control X® software (3D Systems, Rock Hill, SC) (Fig. 6).

Descriptive statistics are presented as mean and range. Comparisons of two continuous and quantitative variables between groups were performed using two-sided paired *t*-tests. Statistical analyses were performed with Minitab® 19 software with a level of significance set at *p* < 0.05.

## Results

### Impingement lesion onto the mobile component chamfer

Under fixed impingement condition, a single contact lesion was observed with the cylindrical geometry whereas a two-point contact lesion was observed with the quadrangular geometry regardless of the surface finishing roughness or head-to-neck ratio (Fig. 6).

Under mobile impingement condition, impingement lesion was circumferential and uniformly spread throughout the entire mobile component chamfer without focalized lesion regardless of the femoral neck geometry or surface finishing roughness (Fig. 6).

### Influence of the femoral neck characteristics on PE damage and wear lesions to the mobile component chamfer

#### Geometry

Under fixed impingement condition, the quadrangular geometry demonstrated significantly higher volumetric wear and impingement deformation depth onto the chamfer when compared to the cylindrical geometry regardless of the surface finishing roughness (*p* = 0.004 to 0.025) (Table 2).

**Table 2:** Influence of the femoral neck geometry on polyethylene wear and damage lesions to the mobile component chamfer (mean [range], 1.0 mc: 1.0 million cycles)

28-mm diameter femoral head	Impingement condition	Surface finishing	Geometry		*p*
			Quadrangular	Cylindrical	
Volumetric wear rate (mm^3^ at 1.0 mc)	Fixed	Rough	8.479 [0–17.456]	0.353 [0–2.059]	0.019
	Fixed	Polished	0.990 [0–2.968]	0.148 [0–0.593]	0.015
	Mobile	Rough	2.164 [0–4.351]	0.358 [0–1.420]	0.283
	Mobile	Polished	0	0	-
Impingement deformation depth (mm at 1.0 mc)	Fixed	Rough	1.091 [0.589–1.441]	0.453 [0.337–0.642]	0.004
	Fixed	Polished	0.735 [0.564–1.024]	0.427 [0.332–0.612]	0.025
	Mobile	Rough	0.359 [0.223–0.573]	0.214 [0.114–0.334]	0.810
	Mobile	Polished	0.252 [0.207–0.348]	0.217 [0.213–0.221]	0.317

However, under mobile impingement condition, no significant difference was detected in volumetric wear and impingement deformation depth onto the chamfer between the quadrangular and cylindrical geometry regardless of the surface finishing roughness (*p* = 0.283 to 0.810) (Table 2).

#### Surface finishing roughness

With the quadrangular geometry, the rough surface finishing produced significantly higher volumetric wear when compared to the polished surface finishing under fixed impingement condition (*p* = 0.009) (Table 3). However, no significant difference in impingement deformation depth onto the chamfer was detected between the rough and polished surface finishing under fixed impingement condition (*p* = 0.201) (Table 3). In addition, no significant difference in volumetric wear and impingement deformation depth onto the chamfer was detected between the rough and polished surface finishing under mobile impingement condition (*p* = 0.372 and 0.253; respectively) (Table 3).

**Table 3:** Influence of the surface finishing roughness of the femoral neck on polyethylene wear and damage lesions to the mobile component chamfer (mean [range], 1.0 mc: 1.0 million cycles)

28-mm diameter femoral head	Geometry	Impingement condition	Surface finishing roughness		*p*
			Rough	Polished	
Volumetric wear rate (mm^3^ at 1.0 mc)	Quadrangular	Fixed	8.479 [0–17.456]	0.990 [0–2.968]	0.009
	Cylindrical	Fixed	0.353 [0–2.059]	0.148 [0–0.593]	0.326
	Quadrangular	Mobile	2.164 [0–4.351]	0	0.372
	Cylindrical	Mobile	0.358 [0–1.420]	0	0.341
Impingement deformation depth (mm at 1.0 mc)	Quadrangular	Fixed	1.091 [0.589–1.441]	0.735 [0.564–1.024]	0.201
	Cylindrical	Fixed	0.453 [0.337–0.642]	0.427 [0.332–0.612]	0.730
	Quadrangular	Mobile	0.359 [0.223–0.573]	0.252 [0.207–0.348]	0.253
	Cylindrical	Mobile	0.214 [0.114–0.334]	0.217 [0.213–0.221]	0.317

With the cylindrical geometry, no significant difference was detected in volumetric wear and impingement deformation depth onto the chamfer between the rough and polished surface finishing regardless of the impingement condition (*p* = 0.317 to 0.730) (Table 3).

#### Head-to-neck ratio

For a given geometry, no significant difference was detected in volumetric wear and impingement deformation depth onto the chamfer between the use of a 28- or 22.2-mm diameter femoral head (*p* = 0.244 to 0.714) (Table 4).

**Table 4:** Influence of the head-to-neck ratio on polyethylene wear and damage lesions to the mobile component chamfer (mean [range], 1.0 mc: 1.0 million cycles)

	Geometry	Impingement condition	Head-to-neck ratio		*p*
			28-mm femoral head	22.2-mm femoral head	
Volumetric wear rate (mm^3^ at 1.0 mc)					
	Quadrangular	Fixed	8.479 [0–17.456]	5.461 [0.700–9.123]	0.528
	Cylindrical	Fixed	0.353 [0–2.059]	0.053 [0–0.160]	0.510
			*p* = 0.019	*p* = 0.024	
Impingement deformation depth (mm at 1.0 mc)					
	Quadrangular	Fixed	1.091 [0.589–1.441]	0.778 [0.541–0.915]	0.244
	Cylindrical	Fixed	0.453 [0.337–0.642]	0.487 [0.443–0.560]	0.714
			*p* = 0.004	*p* = 0.048	

However, regardless of the head-to-neck ratio, the quadrangular geometry demonstrated higher volumetric wear and impingement deformation depth onto the chamfer when compared to the cylindrical geometry (*p* = 0.004 to 0.048) (Table 4).

## Discussion

Along with the description of the third articulation role and reports of potential complications related to long-term wear of the mobile component chamfer such as IPD, general recommendations were advocated in THA with DMC: (1) to remove all fibrotic tissues and osteophytes that could restrict motion at the large articulation, (2) to use a stem with the narrowest and smoothest femoral neck and to avoid skirted femoral head in order to reduce impingement at the third articulation, and (3) to achieve the most favorable head-to-neck ratio with 28-mm diameter femoral head, reserving 22.2-mm diameter femoral head for the smallest sizes of DMC [3–11]. However, to our knowledge, no biomechanical study was dedicated to evaluate PE damage and wear lesions to the mobile component chamfer under mobile and fixed femoral neck impingement conditions, and to determine the optimal characteristics of a stem to be used with DMC. The most important finding of this biomechanical study was that, in a well-functioning DMC with modern designs of implant, the femoral neck characteristics did not affect PE damage and wear lesions to the mobile component chamfer. However, this study also demonstrated that, in case of restricted motion at the large articulation leading to fixed femoral neck impingement onto the mobile component chamfer, higher PE damage and wear lesions to the chamfer were produced by the quadrangular geometry compared to the cylindrical geometry. In addition, with the quadrangular geometry, rough surface finishing of the femoral neck increased volumetric wear of the mobile component chamfer under fixed impingement condition. Contrarily, the surface finishing roughness did not affect PE damage and wear lesions onto the mobile component chamfer with the cylindrical geometry regardless of the impingement condition. Interestingly, for a given femoral neck geometry, no influence of the femoral head diameter (i.e., 28- or 22.2-mm diameter) was observed on PE damage and wear lesions onto the mobile component chamfer. However, regardless of the head-to-neck ratio, the quadrangular geometry demonstrated higher PE damage and wear lesions onto the mobile component chamfer when compared to the cylindrical geometry under fixed impingement condition.

In a systematic review including almost 18,000 THA with DMC, De Martino et al. reported that the IPD rate averaged 0.7% in primary and 1.3% in revision THA [21]. Although less common with latest generations of DMC, IPD is explained by a loss in retentive power for the femoral head related to wear of the chamfer and retentive area as a result of chronic femoral neck/mobile component impingement at the third articulation [3–10]. Mechanisms of IPD were previously described by Philippot et al. throughout a three type classification according to peri-operative findings at the time of revision [6]. Particularly, type II IPD is related to restricted motion of the mobile component at the large articulation with fixed femoral neck impingement leading to focalized PE wear and damage lesions onto the chamfer [6]. In this study, the type II was the most common mechanism of IPD accounting for 51% of the cases with the shortest delay of occurrence compared to type I IPD, which is related to isolated and natural wear of the third articulation in a well-functioning DMC, or type III IPD, which is associated with aseptic loosening of the metal-shell [6]. Several causes that lead to restricted motion at the large articulation have been reported in literature [6, 7, 9, 11, 12, 14]. They were related (1) to implant-specific characteristics in early generation of DMC such as insufficient clearance between the mobile component and the metal-shell at the large articulation, non-optimal design of the mobile component retentive area and chamfer, or wear performance of the first generations of UHMWPE; and (2) to tissue response to THA such as periprostehtic ossifications, arthrofibrosis, or iliopsoas tendon impingement with the mobile component [6, 7, 9, 11, 12, 14]. Therefore, understanding the biomechanical relationship between the femoral neck characteristics and chamfer of the mobile component at the third articulation is of major importance to reduce complications due to wear by selecting optimized implant designs in modern DMC constructs, especially whether restricted motion at the large articulation due to tissue response occurs in vivo with time.

Long-term series evaluating the first generation of Bousquet DMC (Novae®, SERF, Décines, France) implanted from the 1985s to 1990s reported IPD rates up to 4% mainly attributable to the PE quality and/or to the initial designs of metal-shell and mobile component chamfer, but also to the femoral neck characteristics of the Bousquet femoral stem (PF®, Décines, France) [6, 8, 10, 22]. This stem was characterized by a large 16-mm diameter femoral neck made of 316-L staimless steel associated with a non-modular 22.2-mm diameter femoral head [6, 8, 10, 22]. However, during the same period, Vielpeau et al. and Lautridou et al. reported IPD rate of 0.7% at a mean follow-up of 16.5 years with the same first generation of Bousquet DMC when implanted with a 316-L stainless steel Charnley-Kerboull stem (MK3®, Stryker-Howmedica, Herouville, France) that was characterized by a thinner (10-mm diameter) and cylindrical geometry of the femoral neck associated with a non-modular 22.2-mm diameter femoral head [4, 23]. However, the PE mobile components evaluated in these two historical series were made of first-generation UHMWPE with low wear resistance and high potential for oxidation in vivo that could have influenced wear of the chamfer at the third articulation. Similarly, Di Laura et al. evaluated, in a case control study, the PE damage lesions onto the chamfer of retrieved mobile components made of second-generation sequentially annealed highly cross-linked PE due to impingement of two different designs of femoral neck in vivo [11]. This study demonstrated that the occurrence of impingement lesions and severity of PE damages onto the chamfer were higher with the ABG II® femoral neck (Stryker, Mahwah, NJ) that is sharper in geometry with scalloped edge regions and rougher in surface finishing compared to the Rejuvenate® femoral neck (Stryker, Mahwah, NJ) that is uniformly cylindrical in geometry and smoother in surface finishing [11]. Therefore, our biomechanical study strengthened these previous historical and retrieval data and confirmed that the femoral neck characteristics could have major impact on wear of the third articulation, and therefore on the occurrence of potential complications.

This study presented with some limitations. First, several in vivo parameters such as PE oxidation, implant positioning, or motion of mobile component at the large articulation that remains unpredictable in vivo were not considered. Similarly, the clinical consequence of the PE damage and wear lesions observed in this biomechanical study remains unknown. However, considering that cracks could occurred into the mobile component retentive area during the snap-fit of the femoral head, it might be supposed that such impingement lesions onto the chamfer could rationally induce their propagation with time particularly in case of restricted motion at the large articulation [6, 7, 11–14, 24]. Second, the femoral necks were designed and custom-made machined for this study purpose reproducing analog geometries of commercially available implants. Therefore, the tribological aspect of stem cast material (i.e., titanium alloy or stainless steel) was not evaluated in this study. Third, the mobile components were made of second-generation sequentially annealed highly cross-linked PE. Importantly, annealed highly cross-linked PE exhibits crystalline phase and mechanical properties, especially regarding fatigue resistance, that are comparable to conventional UHMWPE while long-term wear resistance is improved by the cross-linking process [25]. Therefore, our results might under-evaluate PE damage and wear lesions due to femoral neck impingement onto mobile components made of conventional UHMWPE that is used in most of the modern DMC constructs [25–27].

In conclusion, this biomechanical study emphasized that the femoral neck characteristics are important to be considered in THA when using a DMC in order to limit PE damage and wear lesions onto the mobile component chamfer due to femoral neck impingement at the third articulation. The main characteristic was found to be the femoral neck geometry over the surface finishing roughness or the head-to-neck ratio, particularly whether restricted motion at the large articulation occurs with time in vivo. Therefore, a cylindrical design of the femoral neck might be favored over a quadrangular design in order to limit those PE lesions leading to potential critical wear at the third articulation and subsequent potential complications such as IPD, even though less frequent with modern generation of DMC.

## Data Availability

All the data and material are saved in a repository file folder and available upon request.
